# The 2024 Outbreak of Parvovirus B19 as a Global Obstetrical Threat Insights From an Obstetrics Referral Center in Northern Italy

**DOI:** 10.1002/jmv.70046

**Published:** 2024-11-12

**Authors:** Beatrice Tassis, Lea Testa, Fulvia Pampo, Simona Boito, Giulia Tiso, Veronica Accurti, Irene Cetin, Nicola Persico

**Affiliations:** ^1^ Department of Maternity and Childhood Fondazione IRCCS Ca’ Granda Ospedale Maggiore Policlinico Milan Italy; ^2^ Department of Clinical Sciences and Community Health Università degli Studi di Milano Milan Italy

**Keywords:** infection, outbreak, Parvovirus b19, pregnancy

## Abstract

A significant increase in Parvovirus B 19 (B19V) infections has been reported in the last months in some European countries. This outbreak could be highly detrimental for pregnant women, considering the capacity of the virus to harm the fetus. However, the magnitude and spread of this outbreak is yet unclear. Evidence from other areas is required and there is the need to focus more on the impact on pregnancy. To this aim, pregnant women with B19V infection who were managed in a referral hospital located in Milan, Northern Italy were reviewed. The primary aim was comparing the number of ascertained cases of B19V infection in the period January–July 2024 to the number of cases recorded in the previous 9 years (2015–2023). Overall, the number of B19V infections markedly increased in the first 7 months of 2024. Until 2023, the number of cases per year were below 7, with no cases reported in 2020–2022, while in the period January‐July 2024, the number raised to 59 (*p* < 0.001). Maternal characteristics and fetal outcomes before and after January 2024 did not differ. In conclusion, Italy is also involved in the ongoing outbreak of B19V infection and pregnant women are exposed to this threat.

Synopsis:

An outbreak of Parvovirus B19 infections is currently ongoing in Italian pregnant women.

## 1 Introduction

Parvovirus B19 (B19V), a single‐stranded round DNA virus, is responsible for “erythema infectiosum,” also known as fifth disease, a common disease in children. Infections during pregnancy can be transmitted vertically to the fetus in about 17%–35% of cases [1] and fetal complications occur in about 10% of cases [2]. Harmful effects to the fetus include fetal anemia, non‐immunenonimmune hydrops fetalis and fetal loss.

Very recent epidemiological evidence suggests an outbreak of B19V. Information emerged from studies performed in France, Spain, Italy and Northern Europe [3, 4, 5, 6, 7]. However, the magnitude of this outbreak and the impact on pregnant women is yet unclear and, for this reason, we deem important to investigate the trend and severity of cases of B19V infection in pregnancy in a large referral hospital located in Milan, Northern Italy.

## 2 Materials and Methods

Outpatient and inpatients data of pregnant women referred to the Fondazione IRCCS Ca’ Granda Ospedale Maggiore Policlinico from January 2015 to July 2024 were reviewed. The primary aim was recording the frequency of ascertained cases of B19V infection in the period January‐July 2024 and comparing these data to the frequencies recorded in the previous 9 years (2015–2023). The secondary aim of the study was comparing the severity of the infections in these two periods. The local Ethics Committee (Lombardia 3) approved the study. Ascertained infection was based on the identification of maternal IgM‐positive serology or detection of DNA PCR in maternal or fetal blood or amniotic fluid. Affected women were counseled on the risk of transmission and complications. All were monitored with serial second level ultrasound scans. Fetal anemia was assessed by pulsed wave Doppler ultrasound evaluation of peak systolic velocity in the middle cerebral artery (MCA‐PSV) [8] and the presence or absence of other signs of infection (hydrops, ascites, skin edema, echogenic bowel, cardiomegaly, placentomegaly). Fetal intrauterine transfusion was planned when the MCA‐PSV exceeded 1.5 times the median. Spontaneous abortion and fetal demise were reported as adverse outcomes.

To assess whether statistically significant changes of the frequency of reported cases occurred over time, we postulated as a null hypothesis a uniform distribution. We assessed whether the observed distribution of cases per year fitted this uniform pattern using the Kolmogorov‐Smirnov uniform test. Despite only 7 out of 12 months were included for 2024, we did a conservative approach considering this period equivalent to 1 year. For the comparisons of the characteristics of the cases diagnosed before and after 2024, we used unpaired Student *t* test, Mann–Whitney test and Fisher Exact test, as appropriate.

## 3 Results

**Figure 1:**
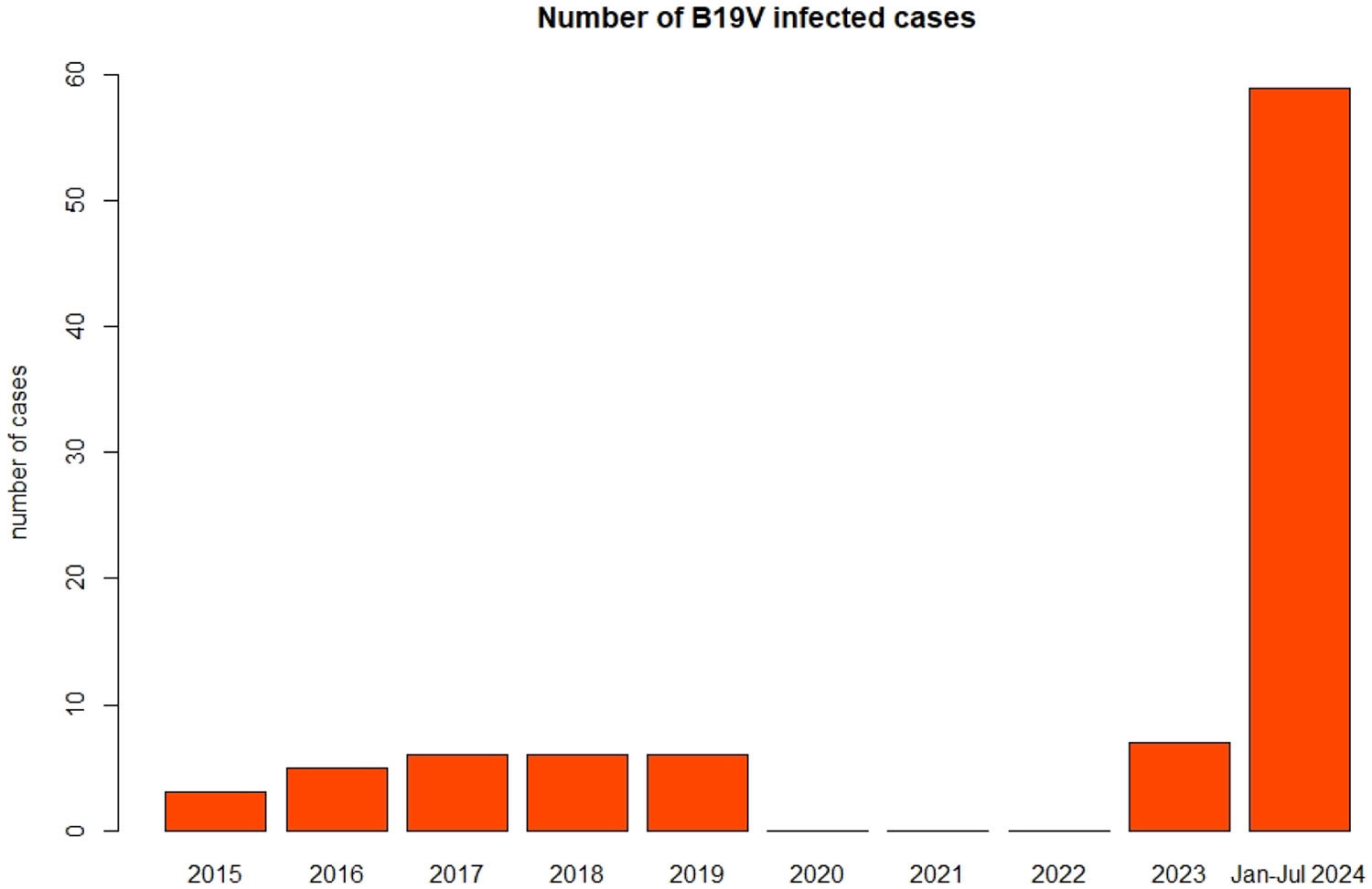
Distribution of the number of pregnant women with Parvovirus B19 infections referred to the Referral hospital in Obstetrics, Fondazione IRCCS Ca’ Granda Ospedale Maggiore Policlinico (Milan, Italy), in the period 2015–2024. The distribution does not fit the null hypothesis of a uniform distribution (corresponding to a theoretical infection rate stable over time) (*p* < 0.001). The distribution does not fit a uniform model also when excluding the 2024 cases because of the absence of cases in 2020‐2022 (*p* = 0.006).

The number of B19V infections markedly increased in the first 7 months of 2024 (Figure 1). Until 2023, the number of cases per year were below 7, with no cases reported in 2020–2022, while in the period January‐July 2024, the number raised to 59. The Kolmogorov‐Smirnov uniform test showed a highly statistical significance (*p* < 0.001). Of note, when excluding 2024 from this analysis, the distribution remained statistically significant (*p* = 0.006) because of the absence of cases during the Covid‐19 pandemic (2020–2022).

Table 1 compares maternal characteristics and fetal outcomes before and after January 2024. One 2024 case was excluded from this analysis because the patient was lost at follow up and therefore did not complete the 12 weeks follow‐up that is necessary to rule out B19V‐related complications. No statistically significant differences were observed between the study periods. The Odds Ratio (95%CI) for ultrasound signs of anemia, definite diagnosis of anemia, intrauterine fetal transfusion and fetal demise in the 2024 periods were 1.40 (0.49–4.31), 2.50 (0.78–9.64), 1.88 (0.51–8.77) and 3.76 (0.41–185.54), respectively.

**Table 1:** Characteristics of the cases of parvovirus B19 infection in pregnancy before and after 2024.

Characteristics	January–July 2024	2015–2023	*p*
	*n* = 58	*n* = 33	
Age (years)	35.2 ± 5.0	34.2 ± 5.0	0.37
Previous deliveries	42 (72%)	25 (76%)	0.81
Gestational age at diagnosis (weeks)	16.7 [11.2:24.5]	15.5 [9.0:21.9]	0.22
Ultrasound signs of fetal anemia	18 (31%)	8 (24%)	0.63
Definite diagnosis of fetal anemia	18 (31%)	5 (15%)	0.13
Intra Uterine fetal transfusions	12 (21%)	4 (12%)	0.40
Adverse outcome (fetal demise)	5 (11%)	1 (3%)	0.39

*Note:* Data are presented as mean ± standard deviation, median [interquartile range] and number (%), as appropriate. Comparisons were made using unpaired Student's *t*‐test, Mann–Whitney test and Fisher Exact test, as appropriate. *p*‐values < 0.05 were considered statistically significant. The total number of cases for the January–July period is 58 rather than 59 because one woman was lost at follow‐up.

## 4 Discussion

4.1

This retrospective study shows that an outbreak of B19V is currently ongoing in Italy, similarly to what reported in Belgium, Denmark, France, Spain, Italy and The Netherlands [3, 4, 5, 6, 7]. The perspective of an Obstetrics Referral Center hampers a broader overview of the infection in the general population, but strengthens the impact on pregnant women, a population at highest risk of clinical consequences. Moreover, the study shows that this outbreak is not characterized by a less aggressive form of infection, the fetal threats being similar to the pre‐2024 period.

An interesting corollary finding is the absence of reported cases in 2020–2022, the period characterized by the Covid‐19 pandemic. These two epidemiological trends may be linked. The COVID‐19 pandemic has led to changes in immunity patterns and hygiene practices that may have altered the seroprevalence of multiple viruses [9, 10, 11], increasing the susceptible host population and its capacity to temper the spread (loss of the herd immunity). Interestingly, a recent survey of our group in pregnant women at term showed that the proportion of women susceptible to B19V infection has significantly increased compared to the past (45% compared to 30%) [12, 13]. This finding may be at first surprising because a 3‐year reduction of infection spread due to Covid‐19 pandemic may seem insufficient for such an impressive impact on the immunity of an adult population. On the other hand, it must be underlined that B19V infection is a typical infection of parous women being infected by their own children. This is evident also in our series, nulliparous women representing a minority (about 25%) in both study periods. In fact, the perturbing effect of the Covid‐19 pandemic on the population susceptibility to viruses may be amplified for B19V because the exposure period for infection in the population is much shorter and overlapped with the Covid‐19 pandemic.

Interestingly, Fappani et al. [5], recently reviewed laboratory findings to monitor measles, rubella and B19V cases in our same region. They also observed an impressive rise in the number of cases, in particular in the adult population. The rise initiated in 2023. Their analysis was based on laboratory findings (they do not present clinical findings) and it did not focus on pregnant women, but indirectly confirm our findings.

Our study raises the question of the potential need for universal screening, at least in this historical phase (up to the time when an equilibrium like in the pre‐Covid‐19 pandemic will be reached). First‐trimester screening could be proposed to identify and appropriately counsel nonimmune subjects on risk factors and hygiene measures to prevent infection [14]. Evidence to support this position is however insufficient. More data and accurate cost–benefit analyses are warranted before claim such change in policy. In the meantime, we recommend strict vigilance for B19V infection in the pregnant population, particularly for parous women. Testing must be promptly performed in case of symptoms in the pregnant women or their offspring.

Some limitations of the study must be recognized. First, and most importantly, we cannot exclude that a growing awareness of B19V among healthcare workers could increase the number of identified cases. Pregnant women could have been tested more frequently than in the past. This could inflate the number of diagnosed cases. In this regard, it would be important to know whether testing for B19 infection has increased in 2024. Indeed, if so, the higher number of identified cases could be ascribable to a higher number of patients tested. Unfortunately, we could not properly address this criticism since affected women were referred to our hospital from the whole region. On the other hand, it must be pointed out that the policy did not change in our hospital (the test is not routinely done), no national or regional alerts advocating routine testing were spread, severity of cases identified before and after 2024 was similar and the observed increase in the number of cases is extremely important. All these elements argue against the critical impact of this confounder. Additional limitations of our study include the retrospective design, the limited sample size hampering a robust comparison of the characteristics of the two groups, and the monocenter recruitment.

In conclusion, Italy seems also involved in the ongoing outbreak of B19V infection and pregnant women are exposed to this threat. This raise seems dramatic, possibly corresponding to a more than 10‐/20‐fold increase. Public health authorities must promptly engage to monitor the situation and consider the possibility to introduce universal screening and global preventive measures, at least up to the end of the surge.

## Author Contributions

Beatrice Tassis, Irene Cetin, and Nicola Persico conceived the study. Lea Testa did the analyses and wrote the first draft of the manuscript. Fulvia Pampo, Simona Boito, Veronica Accurti, and Giulia Tiso collected the data and actively discussed the findings. All the authors extensively revised the manuscript. Beatrice Tassis supervised the whole study.

## Conflicts of Interest

The authors declare no conflicts of interest.

## Data Availability

Research data are not shared.
